# A window into the mind-brain-body interplay: Development of diagnostic, prognostic biomarkers, and rehabilitation strategies in functional motor disorders

**DOI:** 10.1371/journal.pone.0309408

**Published:** 2024-09-26

**Authors:** Marialuisa Gandolfi, Angela Sandri, Sara Mariotto, Stefano Tamburin, Anna Paolicelli, Mirta Fiorio, Giulia Pedrotti, Paolo Barone, Maria Teresa Pellecchia, Roberto Erro, Sofia Cuoco, Immacolata Carotenuto, Claudia Vinciguerra, Annibale Botto, Lucia Zenere, Elisa Canu, Elisa Sibilla, Massimo Filippi, Elisabetta Sarasso, Federica Agosta, Michele Tinazzi, on behalf of the Group Collaborators Consortium

**Affiliations:** 1 Department of Neurosciences, Biomedicine and Movement Sciences, University of Verona, Verona, Italy; 2 Azienda Ospedaliera Universitaria Integrata Verona, Verona, Italy; 3 University of Salerno, Salerno, Italy; 4 Neurological Clinic, AOU San Giovanni di Dio e Ruggi d’Aragona, Salerno, Italy; 5 Department of Neuroradiology, AOU San Giovanni di Dio e Ruggi d’Aragona, Salerno, Italy; 6 Neuroimaging Research Unit, Division of Neuroscience, IRCCS San Raffaele Scientific Institute, Milan, Italy; 7 Vita-Salute San Raffaele University, Milan, Italy; 8 Neurology Unit, IRCCS San Raffaele Scientific Institute, Milan, Italy; 9 Neurorehabilitation Unit, IRCCS San Raffaele Scientific Institute, Milan, Italy; 10 Neurophysiology service, IRCCS San Raffaele Scientific Institute, Milan, Italy; 11 Department of Neuroscience, Rehabilitation, Ophthalmology, Genetics and Maternal Child Health, University of Genoa, Genoa, Italy; Public Library of Science, UNITED STATES OF AMERICA

## Abstract

**Background and aims:**

Functional motor disorders (FMD) present a prevalent, yet misunderstood spectrum of neurological conditions characterized by abnormal movements (i.e., functional limb weakness, tremor, dystonia, gait impairments), leading to substantial disability and diminished quality of life. Despite their high prevalence, FMD often face delayed diagnosis and inadequate treatment, resulting in significant social and economic burdens. The old concept of psychological factors as the primary cause (conversion disorder) has been abandoned due to the need for more evidence about their causal role. According to a predictive coding account, the emerging idea is that symptoms and disability may depend on dysfunctions of a specific neural system integrating interoception, exteroception, and motor control. Consequently, symptoms are construed as perceptions of the body’s state. Besides the main pathophysiological features (abnormal attentional focus, beliefs/expectations, and sense of agency), the lived experience of symptoms and their resulting disability may depend on an altered integration at the neural level of interoception, exteroception, and motor control.

**Methods and materials:**

Our proposal aims to elucidate the pathophysiological mechanisms of FMD through a three-stage research approach. Initially, a large cohort study will collect behavioral, neurophysiological, and MRI biomarkers from patients with FMD and healthy controls, employing eXplainable Artificial Intelligence (XAI) to develop a diagnostic algorithm. Subsequently, validation will occur using patients with organic motor disorders. Finally, the algorithm’s prognostic value will be explored post-rehabilitation in one subgroup of patients with FMD.

**Results:**

Data collection for the present study started in May 2023, and by May 2025, data collection will conclude.

**Discussion:**

Our approach seeks to enhance early diagnosis and prognostication, improve FMD management, and reduce associated disability and socio-economic costs by identifying disease-specific biomarkers.

**Trial registration:**

This trial was registered in clinicaltrials.gov (NCT06328790).

## 1. Introduction

### 1.1 Background and rationale

Functional motor disorders (FMD) are characterized by abnormal movements significantly altered by distractive maneuvers and incongruent with movement disorders seen in typical neurological diseases [1–3]. They represent more than 50% of functional neurological disorders, which have an incidence of 4-12/100.000 per year and a prevalence of 50/100.000 in patients admitted to Neurological clinics [1, 2, 4, 5]. FMD includes highly disabling disorders characterized by movement poverty (weakness and slowness) or movement excess (tremor, dystonia) [5–8]. They can present as isolated motor symptoms or combined with other functional disorders and complain of non-motor symptoms (fatigue, pain), contributing to disability and poor Quality of Life (QoL) [5–8]. The pathophysiology of FMD continues to elude comprehensive understanding [2, 9], and ongoing debate persists regarding its underlying mechanism [2, 9]. Nevertheless, the primary pathophysiological characteristics entail aberrant attentional focus, beliefs/expectations, and a disrupted sense of agency [2, 9–12]. While FMD exhibit physiological correlates with voluntary movement, such as distractibility and resolution with placebo, individuals experiencing FMD perceive these actions as involuntary and beyond their control [2, 9]. According to a predictive coding account [13], the emerging idea is that symptoms are perceptions of the state of the body [2, 9–12]. Therefore, they rely on neural processes that actively sample body information and process it into conscious percepts. The brain uses such percepts to control motor and behavioral responses, producing sensory feedback. Thus, symptoms and disability in FMD may depend on dysfunctions of a specific neural system integrating interoception, exteroception, and motor control [2, 9–12]. Scientific and clinical developments have provided a strong rationale and scientific foundation for identifying specific biomarkers for FMD diagnosis and prognosis [2, 9, 14–17]. It will improve the early identification of FMD patients and maximize the efficacy of multidisciplinary patient management to improve their prognosis [18]. Moreover, it might help detect and quantify functional processes, delineating their underlying neurophysiological mechanisms and understanding how these mechanisms result in functional symptoms, thus overcoming the poor long-term prognosis of FMD [19]. Identifying dependable biomarkers is therefore paramount in enhancing our diagnostic capabilities, enabling the early detection of FMD, and optimizing the effectiveness of multidisciplinary patient management [2, 18, 20].

Furthermore, this endeavor may aid in identifying and quantifying functional processes, elucidating their underlying neurophysiological mechanisms, and providing insights into how these mechanisms manifest as functional symptoms [18]. The current research proposal will fill the existing knowledge gap on FMD diagnosis, pathophysiology, and prognosis by systematically investigating a large cohort of patients with FMD at a behavioral and neural level, exploring the motor, exteroceptive, and interoceptive domains in which hallmarks for FMD have been identified as potential biomarkers of disease. For the first time, the outcomes derived from our experiments will unveil the fundamental biomarkers indispensable for diagnosing FMD, shedding light on crucial disparities between FMD, healthy controls (HC), and organic movement disorders. Both behavioral, neurophysiological and MRI biomarkers will be assessed for their predictive value in determining the diagnosis and prognosis of FMD.

### 1.2 Objectives

The project aims to accomplish three objectives through a structured three-stage methodology involving comprehensive assessments in behavioral, neurophysiological, and MRI domains targeting motor, exteroceptive, and interoceptive functions.

#### 1.2.1 Which biomarker-based diagnostic algorithm for FMD?

The first objective is developing a biomarker-based diagnostic algorithm for FMD by integrating behavioral, neurophysiological, and MRI biomarkers to inspect motor, exteroceptive, and interoceptive domains. These results will distinguish the hallmark biomarkers of FMD diagnosis, highlighting key disparities between FMD and HC.

#### 1.2.2 Is it specific for FMD?

The second objective is to validate the developed biomarker-based diagnostic algorithm by comparing it against patients diagnosed with "organic" motor disorders, assessing its specificity for FMD diagnosis. This endeavor will identify biomarkers specific to FMD diagnosis and elucidate distinguishing features between FMD and patients with "organic" motor disorders.

#### 1.2.3 Could it provide a prognostic insight?

The third objective is to investigate whether the validated biomarkers undergo changes following multidisciplinary rehabilitation training and correlate these changes with shifts in QoL, motor, and non-motor symptoms among FMD patients. This exploration aims to ascertain the potential of these biomarkers in measuring treatment response and providing prognostic insights.

## 2. Methods

### 2.1 Trial design

**Fig 1:**
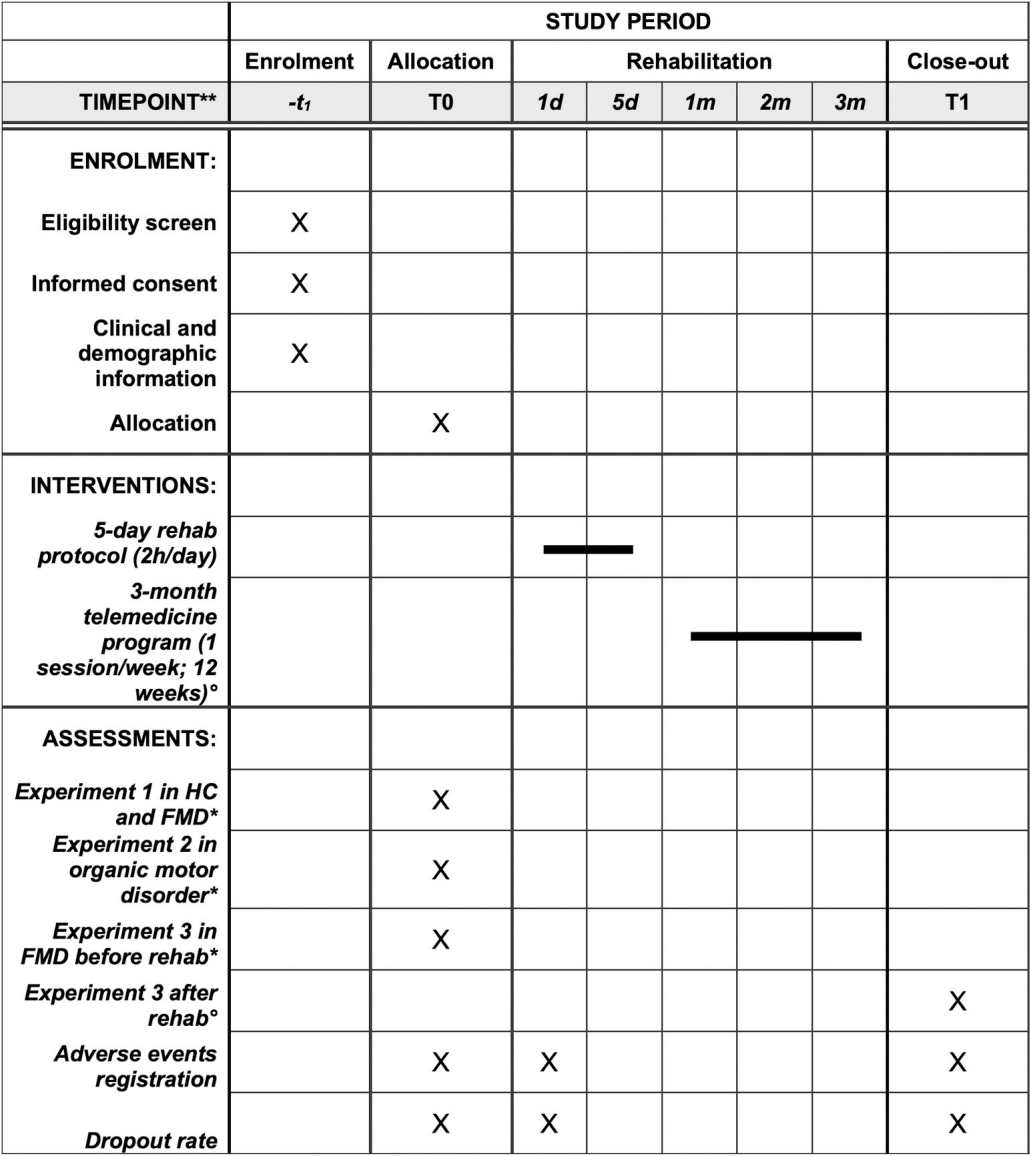
The schedule of enrolment, interventions, and assessments. ° in a sub-group of patients (n = 34); HC, Healthy Control; FMD, Functional Motor Disorder, MRI, Magnetic Resonance Imaging; T0 –assessment before rehabilitation; T1 –assessment at three months Follow-up.

**Fig 2:**
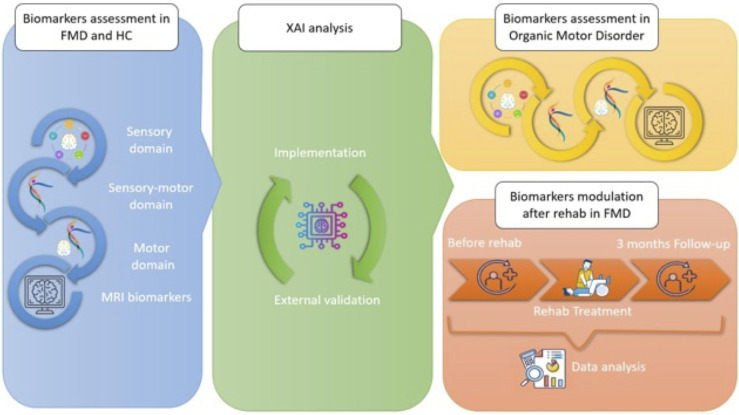
An overview of the organization of the study program. FMD, Functional Motor Disorder; HC, Healthy Control; MRI, Magnetic Resonance Imaging; XAI, eXplainable Artificial Intelligence.

This is an Italian multicenter study of adults with FMD. The project will have three sequential stages, each characterized by a distinct study design tailored to its objectives. Three experiments, each tailored with a distinct study design, will address the specific objectives. Fig 1 shows the schedule of the study procedures for enrolment, interventions, and assessments. Fig 2 depicts the flow diagram of the study.

#### 2.1.1 Experiment 1

It will employ a cross-sectional experimental design, enlisting 150 patients diagnosed with FMD according to the Gupta and Lang diagnostic criteria [21] and 150 HC to achieve objective 1.

#### 2.1.2 Experiment 2

It will employ a cross-sectional design involving 150 patients with "organic" motor disorders to achieve objective 2. This cohort will consist of 50 patients with weakness attributed to peripheral neuromuscular disorders, 50 diagnosed with essential tremor (ET) based on the 2018 consensus criteria [22], and 50 with idiopathic adult-onset dystonia [23].

#### 2.1.3 Experiment 3

It will utilize a prospective longitudinal study design, focusing on a subgroup of FMD patients [24] to achieve objective 3. Evaluations will occur before (T0) and after a 3-month validated rehabilitation protocol (T1) [24, 25].

### 2.2 Study setting and participants

The overarching project involves the collaboration of three Units, each contributing specific expertise to different methodological aspects of the study. Two Units, the Neurological Unit B of the Azienda Ospedaliera Universitaria Integrata (AOUI) of Verona (Italy) and the Neurological Unit of the Azienda Ospedaliera Ruggi D’Aragona of Salerno (Italy), will oversee patient enrollment and assessment, sharing the same methodology. The third unit, Neuroimaging Research Unit, Division of Neuroscience, IRCCS San Raffaele Scientific Institute (Milan, Italy), will analyze MRI data.

#### 2.2.1 Eligibility criteria Experiment 1

150 patients with FMD and 150 age-matched HC will be recruited consecutively. Inclusion criteria for patients will include (1) an age equal to or higher than 18 years and a (2) clinically definite diagnosis of FMD [21]. Exclusion criteria for patients will comprise (1) a Mini-Mental State Examination score equal to or lower than 24/30 [26], (2) physical impairment preventing the signing of informed consent for participation, (3) certified neurological and/or psychiatric comorbidities (such as neuropathy, seizures, and major depression), and (4) contraindications for 3T MRI.

The inclusion criterion for HC will be (1) age higher or equal to 18 years. Exclusion criteria will be (2) Mini-Mental State Examination score lower or equal to 24/30 [26]; (3) physical impairment precluding signing the informed consent for participation; (4) certified neurological and/or psychiatric comorbidities (i.e., neuropathy, seizures, major depression), and (5) contraindications for 3T MRI.

#### 2.2.2 Eligibility criteria Experiment 2

“Organic" motor disorders group will consist of 50 patients with weakness attributed to peripheral neuromuscular disorders, 50 diagnosed with ET based on the 2018 consensus criteria [22], and 50 with idiopathic adult-onset dystonia [23].

Inclusion criteria for these patients will include (1) an age higher or equal to 18 years and (2) a clinically definite diagnosis of “organic” motor disorders represented by weakness due to peripheral neuromuscular disorders, essential tremor, or idiopathic adult-onset dystonia [22, 23]. The same exclusion criteria described for HC and FMD in experiment 1 will be used. Additional exclusion criteria will be (3) a score > 2 on the Tremor Research Group Essential Tremor Rating Assessment Scale, rest tremor and other neurological signs, and current or past exposure to tremorgenic drugs.

#### 2.2.3 Eligibility criteria Experiment 3

Thirty-four individuals with a definite diagnosis of FMD will be software-based randomly selected from the enrolled patients in Experiment 1. Not complying with the rehabilitation program will be an exclusion criterion.

### 2.3 Assessment procedures

A physician specialized in movement disorders will conduct the clinical assessment to determine the patient’s eligibility for inclusion in the project, adhering to the selection criteria at each Unit.

#### 2.3.1 Assessment procedures—Experiment 1

Each patient’s demographic and clinical data will be collected according to the Italian Academy for the Study of Parkinson’s Disease and other Movement Disorders (Accademia LIMPE DISMOV RADAC project), which complied with the General Data Protection Regulation. Clinical motor outcomes will be measured with the objective-rated Simplified Functional Movement Disorders Rating Scale (SFMDRS) [27]; fatigue, pain, anxiety, depression, alexithymia, and Quality of Life (QoL) will be assessed through the Multidimensional Fatigue Inventory Scale (MFI-20), Beck Anxiety Inventory (BAI), Beck Depression Inventory (BDI II), and 12-item Short-Form Health Survey (SF-12), respectively [28–31]. FMD patients and HC will undergo 3T MRI brain scans. With up-to-date tools, cortical thickness metrics, Gray Matter (GM) volumes, and resting-state functional connectivity of sensorimotor and non-motor brain regions will be analyzed [15, 16, 32]. Table 1 reports the potential biomarkers.

**Table 1:** Overview of the development of the biomarkers in the motor, exteroceptive, interoceptive, and cerebral domains.

Domain/Tests	Biomarker
*Motor domain*	
Contingent Negative Variation (CNV)	Amplitude
Pre-pulse inhibition (PPI) of the blink reflex	R2 response magnitude of the blink reflex
Spatiotemporal Gait analysis	Gait speed (cm/s), stride length (cm), stride time variability and swing time variability, swing time (sec), double support time (sec).
Stabilometric assessement	Sway area (mm^2^), length of Center of Pressure (mm), and velocity of CoP displacement in the anteroposterior and mediolateral direction (mm/sec).
*Exteroceptive domain*	
Sensory Attenuation (SA)	The ratio between the matched force and the target force
Tonic vibration reflex (TVR)	The angle of the vibrated arm for TVR and the angle of the tracking arm for the perception of the TVR movement
Somatosensory evoked potentials (SEP)	The late SEP amplitude.
Laser-evoked potentials (LEP)	N2/P2 amplitude
*Interoceptive domain*	
Heartbeat Perception Task (HPT)	objective/subjective HR ratio
*Cerebral*	
T2-weighted fluid-attenuated inversion recovery	Excluding CNS lesions
3D T1-weighted	Morphometric analysis: cortical thickness and grey matter volumes of the primary sensory and motor, executive-attentive, and sense of agency networks and subcortical areas (thalamus, striatum, amygdala).
Resting-state fMRI	Functional connectivity of sensorimotor, executive–attentive, and sense of agency networks.

HR, Heart Rate; CNS, Central Nervous System; fMRI, Functional Magnetic Resonance Imaging

*2*.*3*.*1*.*1 Biomarker development in the motor domain*. Contingent Negative Variation (CNV) is a slow negative EEG wave representing motor preparation and anticipatory attention to a forthcoming imperative cue [33]. It appears to be a good biomarker of FMD [33]. We will record CNV in FMD and HC and predict that it will be absent or reduced in all FMD phenotypes. As a biomarker, we will use CNV amplitude.

Pre-pulse inhibition (PPI) of the blink reflex is a neurophysiological phenomenon to evaluate sensorimotor gating/interaction at the subcortical level [34]. It reflects an automatic, early stage phase of attentional selection processes without awareness [34]. We will record the PPI in FMD and HC and predict that it will be abnormal in all FMD phenotypes. As a biomarker, we will use the magnitude of the R2 response of the blink reflex, which is recorded with EMG.

Gait and postural control analysis will be conducted under both single and dual-task conditions using a spatiotemporal electronic gait analysis system and stabilometric assessment tools (Prokin 252, Tecnobody). Recent evidence regarding posture and gait hints at the benefit of a motor dual-task on posture in FMD [11]. The stabilometric assessment will occur with participants standing on a firm surface without upper limb support. Postural control analysis will involve posture assessment under single, motor, cognitive, and visual fixation conditions. Standardization of foot position and the distance between the two malleoli will be achieved using a V-shaped frame, with the distance between the two malleoli set at 3 cm. The medial borders of the feet will be externally rotated by 12 degrees to the anteroposterior axis [11].

Single-task, motor task, and cognitive dual-task assessments will be conducted under both eyes-open and eyes-closed conditions [11]. Gait analysis will involve walking under single, motor, cognitive, and visual fixation conditions. Each trial will be repeated three times to obtain a mean of gait parameters and reduce bias. Before each task, participants will receive verbal instructions to prioritize either the motor, cognitive, or visual fixation task. S1 outlines the specific conditions for single and dual-task assessments. Recent evidence regarding posture and gait hints at the benefit of a motor dual-task on posture in FMD [11].

*2*.*3*.*1*.*2 Biomarker development in the exteroceptive domain*. Sensory attenuation (SA) is a proxy of sense of agency [35], whereby self-generated actions cause a reduction of sensory perception compared to externally generated actions [35]. We assess SA in FMD and in HC using a force-matching task [35]. As a biomarker of SA, we will use the ratio between the matched force and the target force.

Tonic vibration reflex (TVR) paradigm allows assessing proprioception [12, 36]. We measure the TVR in FMD and HC. We predict that proprioception is abnormal in all FMD phenotypes. As biomarkers, we will use the angle of the vibrated arm for TVR and the angle of the tracking arm for the perception of the TVR movement.

EMG will evaluate somatosensory evoked potential. Reflecting the influence of high-level priors on the processing of bottom-up sensory data. We predict that SEP amplitude is reduced with attention in FMD, reflecting the influence of high-level priors (attention and expectation) on the processing of bottom-up sensory data. As a biomarker, we will use late SEP amplitude.

Laser-evoked potentials (LEP) are a noninvasive measure of the functional status of brain areas involved in nociceptive processing [14, 37, 38]. We assess LEP in FMD and HC when attention is directed towards or away from the stimulated hand. We predict that N2/P2 amplitude is reduced by attention in FMD, reflecting the influence of high-level priors and attention on the processing of bottom-up sensory data. As a biomarker, we will use N2/P2 amplitude.

*2*.*3*.*1*.*3 Biomarker development in the interoceptive domain*. Interoceptive domain will be assessed by Heartbeat Perception Task (HPT) [39], which is a measure of processing of internal bodily signals by the Central Nervous System. We predict a mismatch between the objective and subjective Heat Rate (HR) in FMD, hinting at abnormal prediction. As a biomarker, we will use objective/subjective HR ratio.

*2*.*3*.*1*.*4 Biomarker development in the cerebral domain*. Brain 3 Tesla MRI scans will be acquired in FMD patients and HC. MRI assessment will include T2-weighted fluid-attenuated inversion recovery to exclude CNS lesions, 3D T1-weighted for morphometric analysis (cortical thickness and grey matter volumes) and resting-state fMRI to assess functional connectivity of sensorimotor and non-motor networks [15, 16, 32, 40]. Structural MRI (3DT1 sequences) scans will be analyzed to obtain cortical thickness metrics and grey matter volumes of the primary sensory and motor, executive-attentive, and sense of agency networks and subcortical areas (thalamus, striatum, amygdala). Seed-based resting state fMRI analysis will investigate the functional connectivity of the sensorimotor, executive-attentive, and sense of agency networks.

#### 2.3.2 Assessment procedures Experiment 2

We will assess the same biomarkers identified in the diagnostic biomarker algorithm obtained by the experiment 1 in patients with organic motor disorders [22, 23, 41].

#### 2.3.3 Assessment procedures Experiment 3

We will test the same biomarkers algorithm identified in the diagnostic biomarker algorithm (experiment 1) in FMD before (T0) and after 3 months (T1) of a validated 5-day rehabilitation protocol (2 h/day) within a multidisciplinary etiological framework followed by a home self-management plan under the supervision of a physiotherapist in tele sessions [20, 25, 42–44]. Rehabilitation and home-based self-management are detailed in Table 1.

### 2.4 Statistic plan and data analysis

#### 2.4.1 Sample size calculations

It is based on the simulations performed by Guo et al. 2010 [45]. We will use random forests (RF) owing to their non-parametric nature, contributing to robust performance under settings of varying class conditional biomarker distributions. Assuming a skewed distribution of the dataset and up to 100 neurophysiological and imaging features to include in the statistical analysis, k = 1% (where k is the percentage of biomarkers among n features measured per subject) and effect size for each biomarker < 0.3, we need a sample size of 150 subjects (75 patients with FMD and 75 HC, among all Units) using RF that ensures a statistical power>90%, with a 4-fold cross-validation process. To address concerns regarding the validity of cross-validation-based estimates in relatively small samples, we will pursue an additional external validation based on an independent test set of 150 subjects (75 patients with FMD and 75 HC, among all Units). Finally, the diagnostic biomarker algorithm (experiment 1) will be tested against a population of patients of 150 patients with "organic" motor disorders of similar sample size (50 with weakness due to muscle diseases, 50 with ET, and 50 with dystonia, among all Units). Based on our previous study, for the rehabilitative study Unit 1 will enroll 34 patients, assuming alpha = 0.05, power 90%, and T0-T1 rehabilitation effect size of 0.605 (mean difference 5.52; Standard deviation 9.11) on the primary outcome (S-FMDRS) and a drop-out rate of 10% [20].

#### 2.4.2 Statistical analysis

Ad-hoc models will be identified based on the available data, accounting for type (multi-modal), numerosity, and completeness (missing data). Then, the models will be trained and validated following k-fold cross-validation and tested on a new (unseen) data set to assess their generalization properties. Classical linear methods that are the most widespread in the clinical field will be used for benchmarking. Finally, we will apply eXplainable AI (XAI) analysis to assess feature relevance, and consensus will be assessed under the assumption that this will provide information about the robustness of the outcomes [46–48]. This will also be investigated through association studies with parameters holding clinical relevance. Firstly, we will employ XAI analysis using the dataset from the first recruited 150 subjects (75 patients with FMD and 75 HC). Secondly, external validation will be performed on an independent dataset of features collected on an additional sample of 150 subjects (the remaining 75 patients with FMD and 75 HC). Finally, the biomarkers identified in the diagnostic biomarker algorithm (experiment 1) will be tested against patients with organic neurological disorders recruited. For the rehabilitation sub-study, descriptive statistics will include frequency tables for categorical variables and mean and standard deviation for continuous variables. The normality of data distribution will be checked with the Shapiro-Wilk test. Parametric (paired t-test) or non-parametric tests (Wilcoxon test) will be applied accordingly to compare the means for the two time points (T0, T1). fMRI data will be analyzed using the SPM12 software. One-sample t-tests will evaluate significant mean brain activations during the task. Changes over time will be evaluated using a paired t-test. All the longitudinal data derived from the rehabilitation sub-study will be handled using a complex approach called Gaussian Process Panel Modeling (GPPM). GPPM provides great flexibility because of the many models it can represent. It allows classical statistical inference and machine learning-inspired predictive modeling, with the advantage of obtaining person-specific treatment response predictions [49].

### 2.5 Research ethics approval

The latest revision of the Declaration of Helsinki and the Oviedo Declaration are the basis for the ethical conduct of the study. The study protocol is designed and will be conducted to ensure adherence to the principles and procedures of Good Clinical Practice and to comply with Italian law. The local Ethics Committee has approved the protocol (RC-4201CESC BOdy Mind-Brain-FMD—PNRR-MAD-2022-12376826). This trial was registered in clinicaltrials.gov (NCT06328790).

### 2.6 Consent or assent

At the enrollment, informed consent forms will be made available to all participants engaged in the project, and the patient’s written informed consent before his/her participation in the study will be obtained. Personal information about potential and enrolled participants will be collected, shared, and maintained to protect confidentiality before, during, and after the trial.

### 2.7 Organization of a research project

An overview of the organization of the study program is depicted in Fig 2. This project is structured into three consecutive phases for three experiments, comprising six integrated work packages (WP) to achieve our stated objectives throughout the project’s duration. In the first nine months, the enrollment of patients and HC will be conducted, and clinical and demographic data will be collected following project protocols, along with the acquisition of behavioral, neurophysiological, and MRI biomarkers (WP 1 and 2). By the end of the first year, behavioral, neurophysiological, and MRI biomarkers will be combined and processed separately using a data mining approach (XAI analysis) to develop a computational paradigm to better define their diagnostic value. Validation of the developed biomarkers will be conducted (WP 3).

From months 13 to 22, the resulting biomarkers identified in the diagnostic biomarker algorithm (Experiment 1) will be tested in patients with "organic" motor disorders (WP 4). In addition, the potential modulation of the diagnostic biomarker algorithm (Experiment 1) before and after rehabilitation and clinical improvement of functional motor and non-motor symptoms (NMSs) will be evaluated (WP5). Finally, the final data will be analyzed, disseminated, and communicated by the end of the second year (WP6).

## 3. Results

The data collection phase commences in May 2023. The project is scheduled to conclude within two years, with results expected to be finalized by May 2025.

## 4. Discussion

Our project will uniquely investigate the same cohort of subjects using consistent procedures throughout. This approach ensures a comprehensive and cohesive examination of clinical, neurophysiological, and neuroimaging biomarkers across motor, exteroceptive, and interoceptive domains. By maintaining consistency within the cohort, we aim to yield robust findings that accurately capture the nuances of FMD from their earliest onset. This unified approach enhances the reliability and validity of our results, paving the way for precise diagnostic and prognostic strategies and guiding future intervention trials with greater clarity and confidence. Previously, the development of potential biomarkers included in our project has been investigated individually, often within single or small cohorts of patients, needing a comprehensive overview of their potential. By adopting a cross-sectional approach on the same subjects, we aim to identify potential biomarkers to facilitate early diagnosis of FMD and delineate specific management pathways for future intervention trials, yielding positive socio-economic impact. Specifically, results from experiments 1 and 2 will ascertain which biomarkers serve as hallmarks for FMD diagnosis, elucidating critical distinctions between FMD, HC, and organic movement disorders. Experiment 3 will yield insights into potential clinical and MRI biomarkers predictive of rehabilitation outcomes through applying a diagnostic biomarker algorithm before and after rehabilitation. Finally, our project will furnish specific and sensitive outcome measures while estimating sample sizes for future intervention trials targeting FMD. Addressing challenges such as low data numerosity, data heterogeneity, missing data, and data imbalance represents a pivotal step forward in advancing the state-of-the-art. Utilizing methodologies like transfer learning, multi-task learning, and federated learning will maximize the potential of multi-modal heterogeneous data. Additionally, eXplainable Artificial Intelligence (XAI) will play a crucial role in supporting the definition of numerical biomarkers by identifying the features governing the algorithms, thereby offering clear translational potential. Significant advancements in computational neuroscience, particularly in bodily perception and movement control, present new translational opportunities and insights for assessing the neurobiological integrity of perception and motor control. However, the highly interdisciplinary nature of the project poses challenges, necessitating timely and efficient contributions from professionals with diverse backgrounds ranging from neurology to computer science, cognitive science, neurophysiology, neuroimaging, and physical rehabilitation medicine. Collaboration and integration across these disciplines are essential for effectively addressing the complex issues and driving progress in understanding and managing FMD. Communication and dissemination activities will include the project’s visual identity, public website, social media, videos, and press releases.

## 5. Conclusions

Our proposal will inform the research and clinical community on disease-specific biomarkers to serve for the diagnosis and prognosis of patients with FMD. The proposed approach has significant potential to disentangle some of the poorly understood features of these disorders, potentially providing a platform for more fundamental insights into brain functioning and development of precision medicine approaches in their management. The proposed approach can also provide the clinicians with a set of validated examinations to make a correct early diagnosis. This will improve the management of FMD with a positive impact on the patient’s disability and socio-economic costs of the illness.

## Supporting information

S1 Checklist (DOC)

S1 File Trial study protocol approved EC. (PDF)
