# Supplementary material for: A window into the mind-brain-body interplay: Development of diagnostic, prognostic biomarkers, and rehabilitation strategies in functional motor disorders
Source: PLoS One. 2024 Sep 26;19(9):e0309408. doi: 10.1371/journal.pone.0309408 (PMC11426512; doi:10.1371/journal.pone.0309408)
Supplement: S1 File — (PDF) [file pone.0309408.s002.pdf]

Version 1
Date 21/11/2022

PROJECT TITLE: A window into the mind-brain-body interplay: development of diagnostic, prognostic biomarkers and rehabilitation strategies in functional motor disorders

Short Title: BOdy Mind-Brain-FMD

PROJECT CODE: PNRR-MAD-2022-12376826

PROMOTOR:
USD Malattia di Parkinson e Disordini del Movimento, AOUI Verona;

in collaboration with:
Dipartimento di Neuroscienze, Biomedicina e Movimento – Università di Verona

Principal Investigator:
Prof. Michele Tinazzi
michele.tinazzi@univr.it

Co-Investigator:
Prof.ssa Marialuisa Gandolfi
marialuisa.gandolfi@univr.it

Signature

Data 21/11/2022

Principal Investigator

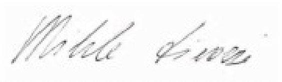

Prof. Michele Tinazzi

INDEX

LIST OF ABBREVIATIONS	PAG 3
ABSTRACT	PAG 4
1. BACKGROUND/STATE OF THE ART	PAG. 5
2. EXPERIMENTAL DESIGN	PAG. 5
3. PROJECT AIMS	PAG. 6
4. SUBJECTS	PAG. 6
5. DISEASE DESCRIPTION	PAG. 7
6. METHODS AND INTERVENTIONS	PAG. 7
7. END POINT	PAG. 9
8. HANDLING AND STORAGE OF ORGANIC SAMPLES (if applicable	PAG. 10
9. DATA MANAGEMENT AND PROCEDURES TO ENSURE DATA CONFIDENTIALITY	PAG. 11
10. STUDY DURATION	PAG. 12
11. SAMPLE SIZE CALCULATION	PAG. 12
12. STATISTICAL ANALYSIS	PAG. 13
13. STUDY LIMITATIONS AND BIAS	PAG. 13
14. ETHICAL ASPECTS AND GOOD CLINICAL PRACTICE	PAG. 13
15. DATA OWNERSHIP E PUBLICATIONS AGREEMENT	PAG. 14
16. BIBLIOGRAPHY	PAG. 14

LIST OF ABBREVIATIONS

FMD: Functional Motor Disorders
HC: Healthy controls
CNS: Central Nervous System
Unit 1: Veneto, AOUI Verona/DAI Neuroscienze/Università di Verona
Unit 2: AOU San Giovanni e Ruggi da Aragona; Università di Salerno/AOU San Giovanni e Ruggi D'Aragona
Unit 3: IRCCS Ospedale San Raffaele; Division of neuroscience
EEG: electroencephalogram
MRI: Magnetic Resonance Image
3T: 3 Tesla
ET: essential Tremor
QoL: Quality of Life
EMG: Electromyography
HPT: objective/subjective Heart Rate HR, ratio
fMRI: function magnetic resonance imaging
WP: work packages
IRFMD: Italian Registry of Functional Motor Disorders
SFMDRS: Simplified Functional Movement Disorders Rating Scale
MFI: Multidimensional Fatigue Inventory Scale
BAI: Beck Anxiety Inventory
BDI-II: Beck Depression Inventory
SF-12: Short-Form Health Survey
XAI: explainable artificial intelligence

ABSTRACT

Functional motor disorders (FMD) are part of the wide spectrum of functional neurological disorders characterized by abnormal movements (functional limb weakness, tremor, dystonia) that are significantly altered by distractive maneuvers and are incongruent with movement disorders seen in specific neurological diseases. FMD have a high prevalence, are still misunderstood, diagnosed with a long delay, and not properly treated, leading to high degrees of disability and poor quality of life with increasing social and economic costs. The old concept of psychological factors as the primary cause (conversion disorder) has been abandoned due to the lack of evidence about their causal role. According to a predictive coding account, the emerging idea is that symptoms and disability in FMD may depend on dysfunctions of a specific neural system integrating interoception, exteroception, and motor control. The idea that underpins our proposal is that FMD symptoms are perceptions of the state of the body. Besides the main pathophysiological features (abnormal attentional focus, beliefs/expectations, and sense of agency), the lived experience of symptoms and their resulting disability may depend on a specific neural system integrating motor, exteroceptive and interoceptive domains. Therefore, dysfunction within this system can cause and sustain motor and non-motor symptoms in FMD. Three-stage research will be conducted. A large cohort of patients with a definite diagnosis of FMD (n=150) and healthy controls (n=150) will be investigated by behavioral, neurophysiological, and MRI tests to collect biomarkers in the motor, exteroceptive and interoceptive domains. Computational modeling of the behavioral, neurophysiological, and MRI biomarkers will be developed through eXplainable Artificial Intelligence (XAI) methods through a data mining approach (machine learning) to implement a diagnostic algorithm biomarker (objective 1). A cohort of patients with "organic" motor disorders (n=75) will undergo the same behavioral, neurophysiological, and MRI tests belonging to the resulting biomarker-based diagnostic algorithm for validation (objective 2). Finally, the modulation of the resulting biomarker-based diagnostic algorithm after rehabilitation and the correlations of motor and non-motor symptoms (NMSs) with clinical improvement will be investigated in a sub-group of patients with FMD to explore the prognostic value (objective 3). Our proposal consists of 6 work packages (WP), all integrated to deliver our stated objectives over the project's lifetime to achieve these objectives. Communication and dissemination activities will include the project's visual identity, public website, social media, videos, and press releases. Our proposal will inform the research and clinical community on disease-specific biomarkers to serve for the diagnosis and prognosis of patients with FMD. The proposed approach has significant potential to disentangle some of the poorly understood features of these disorders, potentially providing a platform for more fundamental insights into brain functioning and development of precision medicine approaches in their management. The proposed approach also can provide the clinicians with a set of validated examinations to make a correct early diagnosis. This will improve the management of FMD with a positive impact on the patient’s disability and socio-economic costs of the illness.

1. BACKGROUND/STATE OF THE ART

FMD is characterized by abnormal movements significantly altered by distractive maneuvers and incongruent with movement disorders seen in typical neurological diseases. They represent more than 50% of functional neurological disorders, which have an incidence of 4-12/100.000 per year and a prevalence of 50/100.000 in patients admitted to Neurological clinics. FMD include highly disabling disorders with poverty (weakness and slowness) or movement excess (tremor, dystonia). They can have isolated or combined motor symptoms and other functional disorders and complain of non-motor symptoms (fatigue, pain), contributing to disability and poor quality of life. The pathophysiology of FMD remains still unknown. According to a predictive coding account, the emerging idea is that symptoms are perceptions of the state of the body. Therefore, they rely on neural processes that actively sample body information and process it into conscious percepts. The brain uses such percepts to control motor and behavioral responses, producing sensory feedback. Thus, symptoms and disability in FMD may depend on dysfunctions of a specific neural system integrating interoception, exteroception, and motor control. Scientific and clinical developments have provided a strong rationale and scientific foundation for identifying specific biomarkers for FMD diagnosis and prognosis. It will improve the early identification of FMD patients and maximize the efficacy of multidisciplinary patient management.

2. EXPERIMENTAL DESIGN

Experimental study without drug or medical device

Experimental design aim 1

This is a cross-sectional study that will involve 150 patients with a clinically definite diagnosis of FMD based on Gupta and Lang diagnostic criteria (1) and 150 matched HC consecutively recruited by Unit 1 (75 patients and 75 HC) and Unit 2 (75 patient s and 75 HC) (WP1). Healthy controls, matched for age and sex, will be recruited through public advertisements. We will develop behavioral, neurophysiological, and MRI biomarkers for each patient and HC in the motor, exteroceptive, interoceptive and cerebral domains (see methods, data collection section). Unit 1 and 2 will perform the same behavioral, neurophysiological, and MRI tests on FMD and HC. 3T MRI data will be processed by Unit 3 to estimate brain structural and functional MRI features of sensorimotor and non-motor networks (WP2). The diagnostic biomarker algorithm through eXplainable Artificial Intelligence (XAI) methods will be developed by Unit 2 (WP3).

Experimental design aim 2

Cross-sectional design on a total of 150 additional patients with "organic" motor disorders (50 patients with weakness due to peripheral neuromuscular disorders; 50 with essential tremor [ET] according to the 2018 consensus criteria (2); 50 with idiopathic adult-onset dystonia) recruited by Unit 1 (75 patients) and 2 (75 patients) (WP4). The same diagnostic biomarker algorithm identified in experiment 1 will be tested in patients with organic motor disorders (WP4) to identify disease-specific hallmarks of FMD. Structural and functional MRI data will be processed by Unit 3. The diagnostic biomarker algorithm through XAI methods will be tested by Unit 2.

Experimental design aim 3

A prospective longitudinal study on 34 out of 75 patients with a definite diagnosis of FMD (aim 1) randomly recruited by Unit 1 will be performed. The diagnostic biomarker algorithm resulting from experiment 1 will be tested in patients with FMD before (T0) and after 3 months (T1) of a rehabilitation protocol validated at Unit 1 (WP5) (13). A physician expert in movement disorders will assess patients at each time point through validated clinical scales to assess motor and non-motor symptoms, depression, anxiety, alexithymia, and QoL

as reported in the Methodologies section. A task-based fMRI paradigm to evaluate functional brain reorganization of sensorimotor and non-motor networks induced by rehabilitation and correlations with clinical improvement will be implemented and performed before rehabilitation (T0) and at 3-month follow- up (T1) (5,6). fMRI data will be analyzed by Unit 3. The diagnostic biomarker algorithm through XAI methods will be tested by Unit 2 before and after rehabilitation.

3. PROJECT AIMS

Specific aim 1

To develop a biomarker-based diagnostic algorithm for FMD by modeling behavioral, neurophysiological, and MRI biomarkers to investigate the motor, exteroceptive and interoceptive domains. Deep learning will be used, and visualization and interpretability methods will be exploited to identify the most sensitive measures. The results obtained will determine which biomarkers are the hallmarks for FMD diagnosis identifying the key differences between FMD and healthy controls.

Specific aim 2

To test the validity of the resulting biomarker-based diagnostic algorithm against patients with "organic" motor disorders to evaluate its specificity for FMD. The results obtained will determine which biomarkers are specific for FMD diagnosis identifying the key differences between FMD and patients with "organic" motor disorders.

Specific aim 3

To explore whether the validated biomarkers change after a multidisciplinary rehabilitation training and correlate them with changes in quality of life and motor and non-motor symptoms of FMD patients to determine their potential role in measuring treatment response and giving prognostic information.

We hypothesize that a composite panel of clinical, neurophysiological, and neuroimaging biomarkers covering motor, exteroceptive and interoceptive domains would be instructive in the diagnosis and prognosis of FMD from the earliest symptoms onset. It would allow the correct diagnosis of these patients early and delineate specific management pathways for future intervention trials with positive socio-economic impact.

4. SUBJECTS

Experimental design 1.

150 patients with a clinically definite diagnosis of FMD based on Gupta and Lang diagnostic criteria (1) and 150 matched HC consecutively recruited by Unit 1 (75 patients and 75 HC) and Unit 2 (75 patient s and 75 HC) (WP1).

Inclusion criteria for patients will be age higher or equal to 18 years and a clinically definite diagnosis of FMD (1). Exclusion criteria for patients will be Mini-Mental State Examination score lower or equal to 24; physical impairment precluding signing the informed consent for participation; certified neurological and/or psychiatric comorbidities (i.e., neuropathy, seizures, major depression); contraindications for 3T MRI.

Inclusion criteria for HC will be age higher or equal to 18 years; Exclusion criteria for HC will be Mini-Mental State Examination score lower or equal to 24; physical impairment precluding signing the informed consent for participation; certified neurological and/or psychiatric comorbidities (i.e., neuropathy, seizures, major depression); contraindications for 3T MRI.

Experimental design 2.

150 additional patients with "organic" motor disorders (50 patients with weakness due to peripheral neuromuscular disorders; 50 with ET according to the 2018 consensus criteria (2); 50 with idiopathic adult-onset dystonia) recruited by Unit 1 (75 patients) and 2 (75 patients) (WP4). ET patients will undergo a neurological assessment by a movement disorder specialist. Patients with idiopathic adult-onset cervical or limb dystonia, either focal or as part of a segmental/multifocal dystonia, will be diagnosed according to published criteria (3). Patients with muscle diseases including idiopathic inflammatory myopathies, sporadic inclusion body myositis and limb-girdle muscular dystrophies will be evaluated according to published criteria (4).

We will assess the same biomarkers identified in the diagnostic biomarker algorithm (experiment 1) in 150 patients with organic motor disorders recruited from Unit 1 and Unit 2 (2-4).
Additional exclusion criteria will be score>2 on the Tremor Research Group Essential Tremor Rating Assessment Scale; rest tremor and other neurological signs; current or past exposure to tremorigenic drugs. Demographic and clinical findings will be collected. Tremors and dystonia severity will be rated using the Fahn-Tolosa-Marin TRS and the Burke-Fahn-Marsden scale. Anxiety, depression, alexithymia, and QoL will be evaluated through the BAI, BDI II, and SF-12.

Experimental design 3.
34 out of 75 patients with a definite diagnosis of FMD (aim 1, Unit 1) will be drawn and tested by the same biomarkers identified in the diagnostic biomarker algorithm (experiment 1) in FMD before (T0) and after 3 months (T1) of a validated 5-day rehabilitation protocol (2 h/day) within a multidisciplinary etiological framework followed by telemedicine program (8,23-25). Patients will be randomly selected from the cohort of patients with FMD recruited in experimental 1 at Unit 1 (n=75), picking a random number from a computer-based list of 75 items. The same inclusion/exclusion criteria of experiment 1 will be used for patients' enrollment. The validated 5-day rehabilitation treatment will be performed by the one physiotherapist participating in the research project. The same physiotherapist will perform the telemedicine program. The rehabilitation treatment will be performed in the rooms of the USD Malattia di Parkinson e Disordini del Movimento, AOUI Verona, under the supervision of the Co-PI (Prof.ssa Gandolfi).kni At T0 and T1, we will perform the same clinical scales and biomarkers included in the biomarker-based diagnostic algorithm (experiment 1) The fMRI task will consist of self-paced alternate dorsal/plantar foot flexion movements (5) with and without cognitive and visual dual-task (6,8).

Drop-out criteria

Patients who will not attend all sessions and/or do not perform all clinical and instrumental evaluations will be considered dropouts. A drop-out rate of 10% is estimated.

5. DISEASE DESCRIPTION

Experimental design 1
Patients with a clinically definite diagnosis of FMD based on Gupta and Lang diagnostic criteria (1) and 150 matched HC consecutively recruited by Unit 1 (75 patients and 75 HC) and Unit 2 (75 patient s and 75 HC) (WP1).

Experimental design 2
Patients with "organic" motor disorders (50 patients with weakness due to peripheral neuromuscular disorders; 50 with ET according to the 2018 consensus criteria (2); 50 with idiopathic adult-onset dystonia) recruited by Unit 1 (75 patients) and 2 (75 patients) (WP4)

Experimental design 3
Patients with a clinically definite diagnosis of FMD based on Gupta and Lang diagnostic criteria (1).

6. METHODS AND INTERVENTIONS

Experimental design 1
Subjects will be assessed at each center by a neurologist specialized in movement disorders. At the enrollment, demographic and clinical data will be collected for each patient according to the Italian Registry of Functional Motor Disorders (IRFMD) (7). Clinical motor outcomes will be measured with the objective-rated Simplified Functional Movement Disorders Rating Scale (SFMDRS) (8); fatigue, pain, anxiety, depression, alexithymia, and Quality of Life (QoL) will be assessed through the Multidimensional Fatigue Inventory Scale (MFI-20), Beck Anxiety Inventory (BAI), Beck Depression Inventory (BDI II), and 12-item Short-Form Health Survey (SF-12) (8) FMD and HC will undergo 3T MRI brain scans (Radiologia BR, AOUI Verona). With up-to-date tools, cortical thickness metrics, GM volumes, and resting-state functional connectivity of sensorimotor and non-motor brain regions will be analyzed (9-12). Assessments will be carried out on three days (the first day clinical assessment; the second day the neurophysiological assessments (Neurologia A, AOUI Verona); the third day the Imaging assessment. MRI brain scan will be performed at the Policlinico GB Rossi.

We will develop behavioral, neurophysiological, and MRI biomarkers for each patient and HC in the motor, exteroceptive, interoceptive and cerebral domains (see methods and interventions). Unit 1 and 2 will perform the same behavioral, neurophysiological, and MRI tests on FMD and HC. 3T MRI data will be processed by Unit 3 to estimate brain structural and functional MRI features of sensorimotor and non-motor networks (WP2). The diagnostic biomarker algorithm through XAI methods will be developed by Unit 2 (WP3). XAI methods will be developed to identify and characterize numerical biomarkers and understand the “why” and the “how” of the machine learning outcomes. XAI is the new frontier of Artificial Intelligence (AI) in that it provides a mean of interpreting the behavior of complex algorithms, enabling their exploitability in the biomedical field. Deep learning will be used, and visualization and interpretability methods will be exploited to develop a biomarker-based diagnostic algorithm. eXplainable AI (XAI) analysis. Details are reported in the Statistical Analysis section.
The implementation of machine learning algorithms will be done using the KNIME analytics platform (v. 4.2.1), widely used in biomedical studies. The software is for research purposes only and not for diagnostic purposes. Data analysis will be anonymized for privacy protection, removing personally identifiable information from data sets (i.e., patients’ number will be changed into a range of values with logical boundaries). In this study, binary supervised classification will be applied with learning derived from decision tree-based (tree-based) and instance-based (instance-based) algorithms. Machine learning input features will include clinical and neurophysiological variables and data extracted from neuroradiological imaging. Feature importance will also be calculated using the Random Forest algorithm to identify the most relevant input features for classification.

Experimental design 2

The same diagnostic biomarker algorithm identified in experiment 1 will be tested in patients with organic motor disorders (WP4) to identify disease-specific hallmarks of FMD. Structural and functional MRI data will be processed by Unit 3. The diagnostic biomarker algorithm through XAI methods will be tested by Unit 2.

Experimental design 3

A physician expert in movement disorders will assess patients at each time point through validated clinical scales to assess motor and non-motor symptoms, depression, anxiety, alexithymia, and QoL as reported in the Methodologies section. A task-based fMRI paradigm to evaluate functional brain reorganization of senso-rimotor and non-motor networks induced by rehabilitation and correlations with clinical improvement will be

implemented and performed before rehabilitation (T0) and at 3-month follow- up (T1) (5,6). fMRI data will be analyzed by Unit 3. The diagnostic biomarker algorithm through XAI methods will be tested by Unit 2 before and after rehabilitation.

Biomarker development in the motor domain:

- Contingent Negative Variation (CNV, amplitude), a slow negative EEG wave representing motor preparation and anticipatory attention to a forthcoming imperative cue (13-14). It will be evaluated by electroencephalogram.
- Pre-pulse inhibition of the blink reflex (R2 response magnitude of the blink reflex), an EMG signal reflecting sensorimotor gating at the subcortical level and an early automatic process (15). It will be evaluated by EMG.
- Single and dual-task posture and gait parameters using stabilometric and gait analyses (16). They will be assessed by spatio-temporal gait analysis and stabilometric assessment.

Biomarker development in the exteroceptive domain:

- Sensory attenuation (SA, ratio between the matched force and the target force), a proxy of sense of agency (17). It will be evaluated by using a custom-made force transducer which measures fingers' strength (Newton).
- Tonic vibration reflex (TVR, angles of the vibrated ì and the tracking arm) to assess proprioception (18-19). It will be evaluated by a physiotherapy vibrator [18].
- Somatosensory evoked potentials (SEP, amplitude) (20) reflecting the influence of high-level priors on the processing of bottom-up sensory data. It will be evaluated by EMG.
- Laser evoked potentials (LEP, N2/P2 amplitude), a non-invasive measure of the functional status of brain areas involved in nociceptive processing (14,21) assessed when attention is directed towards or away from the stimulated hand. It will be evaluated by EMG examination and YAP laser stimulator.
  Biomarker development in the interoceptive domain:
  Heartbeat Perception Task (HPT, objective/subjective Heart Rate HR, ratio) (22), a measure of processing of internal bodily signals by the CNS. Heart rate will be recorded with a Polar wrist-worn (model V800) wireless connected to the chest belt.

Biomarker development in the cerebral domain:

- Brain 3 Tesla MRI scans obtained from FMD, and HC will include T2- weighted FLAIR to exclude CNS lesions; 3D T1-weighted for morphometric analysis (cortical thickness and grey matter volumes); resting-state fMRI to assess functional connectivity of sensorimotor and non-motor networks (9-12).

Rehabilitation protocol

It is a validated 5-day rehabilitation protocol (2 h/day) within a multidisciplinary etiological framework followed by telemedicine program (8,23-25). The in-person 5-days rehabilitation program (2 h/day) will be aimed to re-establish normal movement patterns within a multidisciplinary etiological framework according to a validated rehabilitation protocol for FMD. Treatment will be tailored to the needs of each patient, following general treatment principles: education, exploration of how symptoms affect movement and posture; retraining movements using strategies based on redirection of attention; and development of a self-management plan. Details on the intervention have been described previously (8,23-25). On completion of the 5-days in-person rehabilitation program, the patients will undergo the telemedicine program during one out of the three weekly home-based sessions for 12 consecutive weeks (30 min/session, 1 session/week). It will consist of overground walking at different gait speed, walking backward and sideways, sit-to-stand activities, and squat exercises for lower limb weakness and gait and balance deficits. Bear weight on the hand and straddling a swiss ball will be used for upper limb weakness (31). During each session, the therapist had a video call with the patient via a smartphone or a computer connection according to the patient's availability and preference.

7. END POINT

- From experiments 1 and 2 we will determine which biomarkers are the hallmarks for FMD diagnosis identifying the key differences between FMD, HC, and organic movement disorders.
- From experiment 3, clinical and MRI biomarkers will be tested as predictive for rehabilitation outcomes by applying diagnostic biomarker algorithm before and after rehabilitation.
- We will provide specific and sensitive outcome measures and estimate sample sizes for future intervention trials FMD.

Flowchart.

**Activity**	**Recruitment**	**Visit 1**	**Rahab Intervention**	**Visit 2**
		**T0**		**T1**
Inclusion/exclusion criteria	x			
Consent for participation	x			
Clinical and demographic information	x			
Collecting and developing behavioral, neurophysiological, and MRI biomarkers in Healthy controls and FMD patients*		X		
Collecting and developing behavioral, neurophysiological, and MRI biomarkers in patients with organic motor disorders*		X		
5-day rehab protocol (2h/day) followed by 3-month telemedicine program (1 session/week; 12 weeks)°			X	
Collecting and developing behavioral, neurophysiological, and MRI biomarkers in Healthy controls and FMD patients after rehab°				X
Adverse events		X	X	x
Dropout rate		X	X	X
* Additional procedure				

* Additional procedures: University and research staff (research fellows) will carry out the assessment procedures

° in a sub-group of patients (n=34)

8. HANDLING AND STORAGE OF ORGANIC SAMPLES (if applicable)

Not applicable

9. DATA MANAGEMENT AND PROCEDURES TO ENSURE DATA CONFIDENTIALITY

The Promoter agrees to comply with the Privacy Laws (as defined below), including those areas relating to security measures and confidentiality. The Promoter warrants, on his own behalf and on behalf of the Investigator, to be fully aware of all obligations arising from any applicable legislation relating to medical professional secrecy and the protection of patients' personal data, including but not limited to the EU Regulation 2016/679, the Privacy Code (D.Lgs 196/03, s.m.i), the provisions, guidelines and general authorisations of the Italian Data Protection Authority (collectively "Privacy Laws").

The Promoter undertakes that all its personnel involved in the conduct of the study will comply with the Privacy Laws and the Promoter's instructions regarding the protection of personal data, including aspects of data security and confidentiality. This obligation includes, for example: (i) providing the patient involved in the study with a complete privacy policy in accordance with the law (in particular, EU Regulation 2016/679, Legislative Decree 196/2003 as amended, as well as the above-mentioned Guidelines of 24 July 2008); (ii) obtaining the patient's written informed consent prior to his/her participation in the study; (iii) respecting the privacy rights of each data subject as established by the applicable Privacy Laws; (iv) taking all appropriate physical, logical, organisational, technical and IT measures in compliance with the applicable Privacy Laws.

Due to the particular sensitivity of the data processed in the firm, specific technical measures have been adopted to increase the level of data security, without prejudice to any other minimum measures. This, with particular reference to the operations of recording with electronic and/or paper instruments the data of the persons involved in the study at the test centre, to their transfer by telematic means to subjects who carry out, on behalf of the latter, the validation and statistical processing of the data, as well as the management of the same database. In connection with these processing operations, the promoter has taken appropriate measures to ensure the protection of the recorded data from the risks of unauthorised access, theft or partial or total loss of the paper documents, storage media or portable or fixed processing systems.

A username and password are required to access the computers where data is entered. In addition, access to the database in order to start a data entry session is protected by username and password.

The following precautionary measures are taken to guarantee data confidentiality and to prevent data manipulation and loss:

- Access to the data is restricted to authorised members only. Authorised members are: Researcher in charge of research, Principal Investigator, examining physician.
- The network is protected by firewalls
- The Internet connection is encrypted with a digital certificate (SSL technology)
- The database is located on a server, protected by a password that is changed periodically.
- Access to the database is password protected and is only accessible to the persons responsible for the centre.
- Regular back-ups are performed.

Paper materials relating to clinical evaluations will be stored in cabinets, the keys to which will only be in the possession of persons authorised by the persons in charge of the study site.

The promoter has adopted a centralised database at the promoter or others who perform the subsequent validation and statistical processing of the data.

The promoter guarantees that it will require the adoption of secure communication protocols for the transmission of any paper documentation.

Finally, it is specified that with specific reference to the database, suitable authentication and authorisation systems are adopted for the persons in charge according to the roles and requirements of access and processing and suitable procedures for the periodic verification of the quality and consistency of the authentication credentials and authorisation profiles assigned to the persons in charge of processing.

The Investigator will appropriately disassociate patient identification data from the data relating to the results of the trial (e.g. by anonymising such data by identifying the results with a randomly generated alphanumeric code) so that only pseudo-anonymised data will be processed by the Promoter.

The Promoter will allow access to clinical data (including medical records) and any other information that may be relevant to the study, always in accordance with applicable Privacy Laws and respecting the security measures and confidentiality of the data.

Within the limits of the information provided to the patients of the trial, the personal data of these subjects will be accessible only to employees, collaborators, monitors and auditors of the Promoter, and/or by the competent authorities in the exercise of their functions.
The Promoter and the Investigator undertake to inform each trial patient clearly and completely about the way in which their personal data will be processed prior to their participation in the trial, in accordance with the applicable Privacy Laws.
Prior to the acquisition of the data, the Investigator agrees to make appropriate disclosures to each patient regarding the nature, purpose, results, consequences and risks of the study prior to that patient's participation in the study. Prior to the patient's enrolment, the Investigator or his authorised delegate will obtain in writing the patient's informed consent: (a) to participate in the study; (b) to the communication of the relevant confidential information; (c) to the processing of personal data; (d) to the transfer of the documentation containing the patient's personal data, including sensitive health data, to the Promoter and/or to the competent authorities and/or to other institutions, including those outside the European Union, in compliance with the laws and in accordance with the applicable Privacy Laws.
The Promoter undertakes to keep the original paper documentation (e.g. informed consent) for at least 7 years in compliance with dl 200/2007.

10. STUDY DURATION

Total duration of the study: 24 months (including data analysis)

This project includes three consecutive phases, for 6 work packages (WP) integrated to deliver our stated objectives over the project's lifetime. During the first 9 months of the project, Unit 1 and Unit 2 will recruit patients and HC, collect clinical and demographic data according to the project protocols, collect and develop behavioral, neurophysiological, and MRI biomarkers. MRI scans will be obtained from the Unit 1 and 2 and sent to Unit 3 for analysis (WP1, WP2). From 10 to 12 months, the behavioral, neurophysiological, and MRI biomarkers will be combined and processed separately by means of a data mining approach (XAI analysis) to develop a computational paradigm aiming at better defining their diagnostic value and then to validate them for Unit 1 and 2 (WP3). From 13 to 22 months, Unit 1 and 2 will assess the resulting biomarkers identified in the diagnostic biomarker algorithm (experiment 1)in FMD in a cohort of patients with "organic" motor disorders (weakness due to peripheral neuromuscular disorders, essential tremor, and idiopathic adult-onset dystonia); and the Unit 1 will evaluate the possible modulation of the diagnostic biomarker algorithm (experiment 1) before and after rehabilitation and clinical improvement of functional motor and NMSs symptoms. Unit 3 will provide MRI biomarkers for patients with "organic" motor disorders (WP4) and analyze task-based fMRI before and after rehabilitation (WP5). From 23 to 24 months, we will analyze, disseminate, and communicate the final data (WP6). Dissemination and communication (WP 6) will include the project's visual identity (logo, packaging, website, and even your email templates), public website, leaflets and flyers, social media, videos, and press releases. Dissemination activities will maximize the impact of research results in the public domain, such as the scientific community, stakeholders, policymakers, through an open science framework and patient's charity. Primary and secondary results will be exploited and disseminated through scientific papers, conferences, talks, workshops.

11. SAMPLE SIZE CALCULATION

Sample size calculations are based on the simulations performed by Guo et al. 2010 (26). We will use Random Forests (RF) owing to their non-parametric nature that contributes to robust performance under settings of varying class conditional biomarkers distributions. Assuming a skewed distribution of the dataset and up to 100 neurophysiological and imaging features to include in the statistical analysis, k=1% (where k is the percentage of biomarkers among n features measured per subject) and effect size for each biomarker < 0.3, we need a sample size of 150 subjects (75 patients with FMD and 75 HC, among all Units) using RF that ensures a statistical power>90%, with a 4-fold cross-validation process. To address concerns regarding the

validity of cross-validation-based estimates in relatively small samples, we will pursue an additional external validation based on an independent test set of 150 subjects (75 patients with FMDs and 75 HC, among all Units). Finally, the diagnostic biomarker algorithm (experiment 1) will be tested against a population of patients of 150 patients with "organic" motor disorders of similar sample size (50 with weakness due to muscle diseases, 50 with ET, and 50 with dystonia, among all Units). Based on our previous study, for the rehabilitative study Unit 1 will enroll 34 patients, assuming alpha= 0.05, power 90%, and T0-T1 rehabilitation effect size of 0.605 (mean difference 5.52; Standard deviation 9.11) on the primary outcome (SFMDRS) and a drop-out rate of 10%(8).

12. STATISTICAL ANALYSIS

eXplainable AI (XAI) analysis. First, ad-hoc models will be identified based on the available data, accounting for type (multi- modal), numerosity, completeness (missing data). Then, the models will be trained and validated following k-fold cross- validation and finally tested on a new (unseen) set of data to assess their generalization properties. Classical linear methods that are the most widespread in the clinical field will be used for benchmarking. Finally, we will apply XAI methods for assessing feature relevance, and consensus will be assessed under the assumption that this will provide information about the robustness of the outcomes (27-29). This will also be investigated through association studies with parameters holding clinical relevance.

Firstly, we will employ XAI analysis using the dataset from the first recruited 150 subjects (75 FMD and 75 HC). Secondly, external validation will be performed on an independent dataset of features collected on an additional sample of 150 subjects (75 FMD and 75 HC). Finally, the biomarkers identified in the diagnostic biomarker algorithm (experiment 1) will be tested against patients with organic neurological disorders recruited by Unit 1 and 2.

For the rehabilitation sub-study, descriptive statistics will include frequency tables for categorical variables and mean and standard deviation for continuous variables. The normality of data distribution will be checked with the Shapiro-Wilk test. Parametric (paired t-test) or non-parametric tests (Wilcoxon test) will be applied accordingly to compare the means for the two-time points (T0, T1). fMRI data will be analyzed using the SPM12 software. One-sample t tests will be used to evaluate significant mean brain activations during the task. Changes over time will be evaluated using a paired t test. All the longitudinal data deriving from the rehabilitation sub-study will be handled with a complex approach named Gaussian Process Panel Modeling (GPPM). GPPM provides great flexibility because of the large number of models it can represent. It allows classical statistical inference as well as machine learning inspired predictive modeling, with the advantage of obtaining person-specific predictions of treatment response (30).

13. STUDY LIMITATIONS AND BIAS

Challenges like low data numerosity, data heterogeneity, missing data, data imbalance are open issues whose solution would lead a step forward in the state-of-the-art. The use of methods-like transfer learning, multi-task learning, and federated learning will fully exploit the potential of multi-modal heterogeneous data. In addition, XAI will substantially support the definition of numerical biomarkers by identifying the features ruling the algorithms holding a clear translational potential (27-29). Significant developments in computational neuroscience, especially about bodily perception, and movement control, allow new translational opportunities and insights to assess the neurobiological integrity of perception and motor control. Challenging aspects are the highly interdisciplinary nature of the project that requires a timely and efficient contribution of professionals with different backgrounds, from neurology to computer science, cognitive science, neurophysiology, neuroimaging, and physical rehabilitation medicine.

14. ETHICAL ASPECTS AND GOOD CLINICAL PRACTICE

The latest revision of the Declaration of Helsinki as well as the Oviedo Declaration are the basis for the ethical conduct of the study. The study protocol is designed and will be conducted to ensure adherence to the principles and procedures of Good Clinical Practice and to comply with Italian law as described in the following documents and accepted, by signature, by the investigators of the study:

1. ICH Harmonized Tripartite Guidelines for Good Clinical Practice 1996.

2. Directive 91/507/EEC, The Rules Governing Medicinal Products in the European

Community.

3. D. L.vo n.211 del 24 giugno 2003.

4. D. L.vo n.200 6 Novembre 2007.

5. D.M. 21 Dicembre 2007.

6. Determinazione AIFA 20 Marzo 2008.

All essential clinical documents will be kept to prove the validity of the study and the integrity of the data collected.

15. DATA OWNERSHIP E PUBLICATIONS AGREEMENT

Study promotor and Research staff.

16. BIBLIOGRAPHY

1. Gupta A, Curr Opin Neurol 2009;22:430-6.
2. Bhatia KP, Mov Disord 2018;33:75-87.
3. Albanese A, Mov Disord 2013;28:863-73.
4. Engel AG, Myology, vol.II, 3rd ed. New York:McGraw-Hill;2004 5. Piramide N, J Neurol 2020;267:1116-26.
6. Cojan Y, Neuroimage 2009;47:1026-37.
7. Tinazzi M, Mov Disord Clin Pract 2020a;7:920-9.
8. Gandolfi M, ... Tinazzi M. NeuroRehabilitation. 2021;48(3):305-314.
9. Tomic A, Agosta F, ..., Filippi M. Mol Psychiatry. 2020 Dec;25(12):3350-3359.
10. Canu E, Agosta F, ..., Filippi M. Hum Brain Mapp. 2020 Aug 1;41(11):3059-3076.
11. Piramide N, ..., Agosta F. J Neurol. 2021 Nov 12. doi: 10.1007/s00415-021-10879-x.
12. Landolfi A, ..., Barone P, Amboni M. Curr Med Chem. 2021;28(32):6548-6568.
13. Teodoro T Eur J Neurol 2020;27:985-94.
14. Lorenz J Neurophysiol Clin 2003;33:293-301.
15. Hanzlikova Z Mov Disord 2019;34:1022-30
16. Gandolfi M, ..., Tinazzi M. Gait Posture. 2021 Jul;88:286-291.
17. Parees I, Brain 2014;137:2916-21.
18. Tinazzi M. Parkinsonism Relat Disord. 2021 Jun;87:1-6.
19. Grunewald RA Brain 1997;120:2179-85.
20. Fiorio M,...Tinazzi M. Neuroscience 2012;217:96-104.
21. Morgante F,...Tinazzi M. Mov Disord 2018;33:1340-8.
22. Schandry R. Psychophysiology. 1981;18(4):483-488.
23. Nielsen G, J Neurol 2015;262:674-81.
24.Nielsen G, J Neurol Neurosurg Psychiatry 2017;88:484-90.
25. Demartini B,...Tinazzi M. Park Relat Disord 2020;76:108-11.
26. Guo, Y BMC Bioinformatics 2010;11,447.

27. Boscolo Galazzo I IEEE Signal Processing Magazine 2021.10.1109/MSP.2021.3126573.
28. Cruciani F J Neural Eng. 2021;18(4).
29. A. Salih IEEE 34th International Symposium on Computer-Based Medical Systems, 2021, pp. 492-497.
30. Gaussian Process Panel Modeling-Machine Learning Inspired Analysis of Longitudinal Panel Data. Front. Psychol., 19 March 2020 | https://doi.org/10.3389/fpsyg.2020.00351
31. Gandolfi M, Sandri A, Geroin C, et al. Improvement in motor symptoms, physical fatigue, and self-rated change perception in functional motor disorders: a prospective cohort study of a 12-week telemedicine program. *J Neurol*. 2022;269(11):5940-5953. doi:10.1007/s00415-022-11230-8
